# Evaluation of Epirubicin in Thermogelling and Bioadhesive Liquid and Solid Suppository Formulations for Rectal Administration

**DOI:** 10.3390/ijms15010342

**Published:** 2013-12-31

**Authors:** Yu-Li Lo, Yijun Lin, Hong-Ru Lin

**Affiliations:** 1 Department of Biological Sciences and Technology, National University of Tainan, Tainan 700, Taiwan; E-Mail: lohograce@gmail.com; 2 Department of Chemical and Materials Engineering, Southern Taiwan University of Science and Technology, Tainan 710, Taiwan; E-Mail: hrlin@stust.edu.tw

**Keywords:** hydrogel, pluronic, polyacrylic acid, epirubicin, colon, suppository

## Abstract

Temperature sensitive Pluronic (Plu) and pH-sensitive polyacrylic acid (PAA) were successfully mixed in different ratios to form *in situ* gelling formulations for colon cancer therapy. The major formulations were prepared as the liquid and solid suppository dosage forms. Epirubicin (Epi) was chosen as a model anticancer drug. *In vitro* characterization and *in vivo* pharmacokinetics and therapeutic efficacy of Epi in six Plu/PAA formulations were evaluated. Our *in vitro* data indicate that Epi in Plu 14%/PAA 0.75% of both solid and liquid suppositories possess significant cytotoxicity, strong bioadhesive force, long-term appropriate suppository base, sustained release, and high accumulation of Epi in rat rectums. These solid and liquid suppositories were retained in the upper rectum of Sprague-Dawley (SD) rats for at least 12 h. An *in vivo* pharmacokinetic study using SD rats showed that after rectal administration of solid and liquid suppositories, Epi had greater area under the curve and higher relative bioavailability than in a rectal solution. These solid and liquid suppositories exhibited remarkable inhibition on the tumor growth of CT26 bearing Balb/c mice *in vivo*. Our findings suggest that *in situ* thermogelling and mucoadhesive suppositories demonstrate a great potential as colon anticancer delivery systems for protracted release of chemotherapeutic agents.

## 1. Introduction

Colorectal cancer is the third most lethal cause of cancer death. Tumor resection, chemotherapy, and radiotherapy are major treatment types for colorectal cancer. Numerous patients need to receive chemotherapy after surgery. The most common routes for chemotherapy are injection and oral administration. However, oral route may have the drawbacks of substantial gastrointestinal (GI) and hepatic first-pass effect [1]. On the other hand, when administered intravenously, some anticancer drugs, such as epirubicin, may cause damage to the vein into which it is injected [2]. The rectal route for drug administration possesses the advantage of the decreased side effects by avoiding both oral unpleasant taste and reducing GI irritation [3]. However, the conventional solid suppository has the drawbacks of patient discomfort, leading to decreased patient compliance. For the target-delivery of anti-colon cancer drugs, ideal suppository should possess the mucoadhesive characteristic and remain in the lower rectum for a long period of time to reduce the first pass effect [4]. We thus use a pH- and thermo-sensitive *in situ* gelling liquid suppository system with suitable gel strength and bioadhesive force to solve these problems. Moreover, a non-mucoadhesive solid suppository has the weakness, because such a suppository quickly melts in the rectum, leading to rapid release of the carried drugs to undergo the first-pass effect [5,6]. Furthermore, a traditional solid suppository base has the disadvantages of fast melting or softening in the rectum without a sustained-release function for the carried drugs [7]. Thus, we also designed a cocoa butter-based solid suppository with an enclosed mucoadhesive, pH- and thermo-gelling system for delivering drugs.

**Figure 1.**
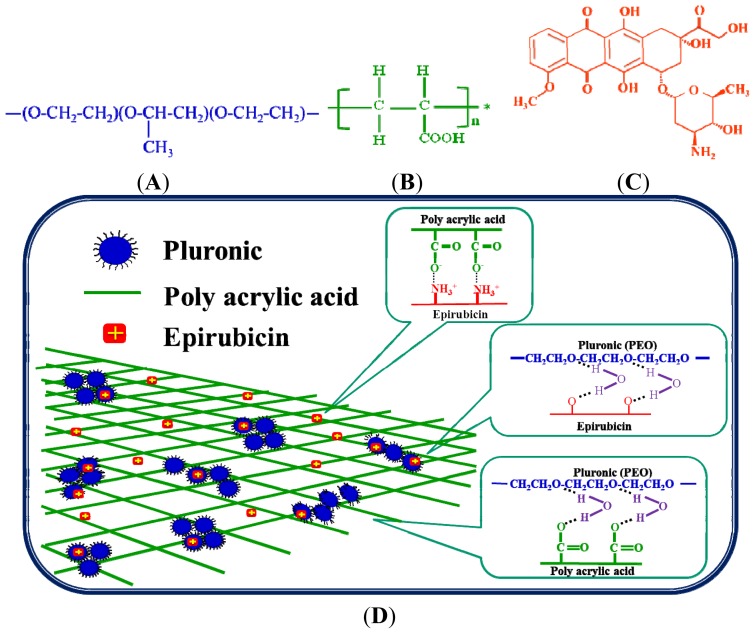
Chemical structure of (**A**) pluronic (Plu); (**B**) polyacrylic acid (PAA); (**C**) epirubicin (Epi); (**D**) semi-interpenetrating network structure of the Plu/PAA/Epi hydrogel.

Hydrogels are three-dimensional polymers and are widely used in controlled drug delivery [8,9]. Pluronic (Plu.; Figure 1A), one of the trademarks of poloxamers, is composed of polyethylene oxide (PEO) and polypropylene oxide (PPO) as PEO–PPO–PEO triblock copolymers. This polymer has been widely used in the preparation of liquid suppository [3,5,7]. It is usually considered as nontoxic and has thus been applied in localized drug delivery such as intramuscular, intraperitoneal, and subcutaneous injections that slowly and continuously releases the drugs to the tumor and surrounding tissue [10–13]. Pluronic would arrange into micelles when temperature is above 25 °C and thus change from liquid to gel state [10]. Polyacrylic acid (PAA; Figure 1B) is a hydrophilic pH-sensitive hydrogel. This polymer is biodegradable by GI enzymes [14]. It is a mucoadhesive polyelectrolyte with COOH on its side chain [15] and deprotonated into carboxylate ions when the pH is greater than its pKa (4.75) [16]. Here, we used PAA to strengthen the drawbacks of Pluronic, such as weak gel strength, rapid erosion, and non-biodegradability [17]. Additionally, Pluronic may improve the disadvantages of PAA, *i.e*., a high glass transition temperature and high water solubility [18].

Epirubicin (Epi; Figure 1C) is an amphiphilic anthracycline anticancer drug. It exhibits high antitumor activity and has been widely applied in chemotherapy of breast, gastric, colorectal, and ovarian cancers [2,19–21]. In our previous studies, a liquid suppository composed of Plu and PAA for site-targeting delivery of oxaliplatin was evaluated [7]. We also developed a Pluronic/PAA system for the oral delivery of Epi [22,23], and our results suggest that this system is a convenient and effective intestinal dosage form. In this study, we developed an *in situ* gelling liquid suppository formulation, which is composed of Pluronic and PAA mixture (Table 1), for rectal administration of Epi. Plu and PAA can be administered in a liquid form into the anus, and changed from sol to gel state instantly in colorectal physiological condition (pH 7.4 and 37 °C). The Plu and PAA system retains mucoadhesive, thermo-, and pH-sensitive characteristics with the potential for improving the site-targeting delivery of Epi. The schematic diagram of forming Plu/PAA/Epi hydrogel is shown in Figure 1D. Furthermore, a cocoa butter-based solid suppository with the enclosed Plu and PAA mixture was also prepared for delivering Epi. This system melts, instantly gels in the rectum, and adheres to the mucous membrane for sustained release of Epi. In this study, we focused on the *in vitro* investigation of bioadhesive force, cytotoxicity, drug permeation and accumulation, as well as structural stability of these suppository formulations. In addition, we also assessed *in vivo* localization, pharmacokinetic evaluation, and antitumor efficacy of these suppositories in Sprague-Dawley (SD) rats and mouse colon adenocarcinoma CT26 bearing Balb/c mice.

## 2. Results and Discussion

### 2.1. Results

#### 2.1.1. Cell Growth Inhibition Assay

**Figure 2.**
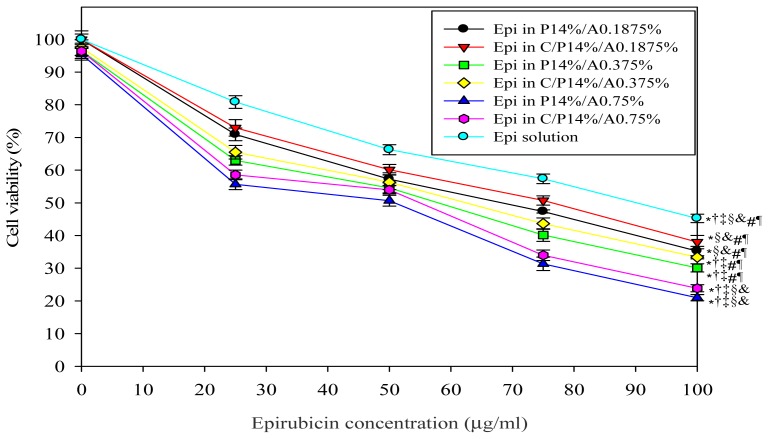
*In vitro* cytotoxic effects of Epi in liquid and solid suppositories on CT26 cells. These suppositories consist of different Plu/PAA ratios with various Epi concentrations at 0, 25, 50, 75, and 100 μg/mL. C represents cocoa butter; P for Plu; A for PAA. * *p* < 0.05 compared with Epi solution; ^†^ *p* < 0.05 compared with Epi in P14%/A0.1875%; ^‡^ *p* < 0.05 compared with Epi in C/P14%/A0.1875%; ^§^ *p* < 0.05 compared with Epi in P14%/A0.375%; ^&^ *p* < 0.05 compared with Epi in C/P14%/A0.375%; ^#^ *p* < 0.05 compared with Epi in P14%/A0.75%; ^¶^ *p* < 0.05 compared with Epi in C/P14%/A0.75%.

In this study, we used MTT assay to evaluate the cytotoxic effect of Plu/PAA formulations with various Epi concentrations in the presence of cocoa butter (0.5 g; solid suppository) or absence (liquid suppository) on growth inhibition of CT26 cells. The formulations without Epi had no significant cytotoxicity on CT26 cells, indicating that these components were non-toxic to CT26 cells (Figure 2). As Epi concentrations in liquid or solid suppository formulations were increased from 25 to 100 μg/mL, CT26 cell viability % was decreased dose-dependently (Figure 2). Both liquid and solid suppository formulations demonstrated similar effect on cell viability at the same Plu/PAA concentration (*p* > 0.05). Furthermore, our results also suggested that 50 μg/mL of Epi loaded in a Plu 14%/PAA 0.75% hydrogel either in liquid or solid suppository formulations showed about 50% inhibition of CT26 cell growth (Figure 2). Thus, this concentration of Epi was selected for the following investigations.

#### 2.1.2. Determination of Bioadhesive Force

We measured the bioadhesive force of both the liquid and solid suppository of Formulations 1–6 on the rectal membrane. The data demonstrated in Table 2 shows that the bioadhesive force of the hydrogel of Formulation 1 is significantly higher than that of the hydrogels of Formulations 2 and 3 (both *p* < 0.005). Cocoa butter has the bioadhesive force of 5.94 ± 0.57 g, which is between the value of Formulations 1 and 2. The addition of cocoa butter in the formulations increased the bioadhesive force. As listed in Table 2, the value of the bioadhesive force of Formulation 4 was higher than that of Formulation 1; Formulation 5 > Formulation 2; Formulation 6 > Formulation 3.

#### 2.1.3. *In Vitro* Drug Release Study

**Figure 3.**
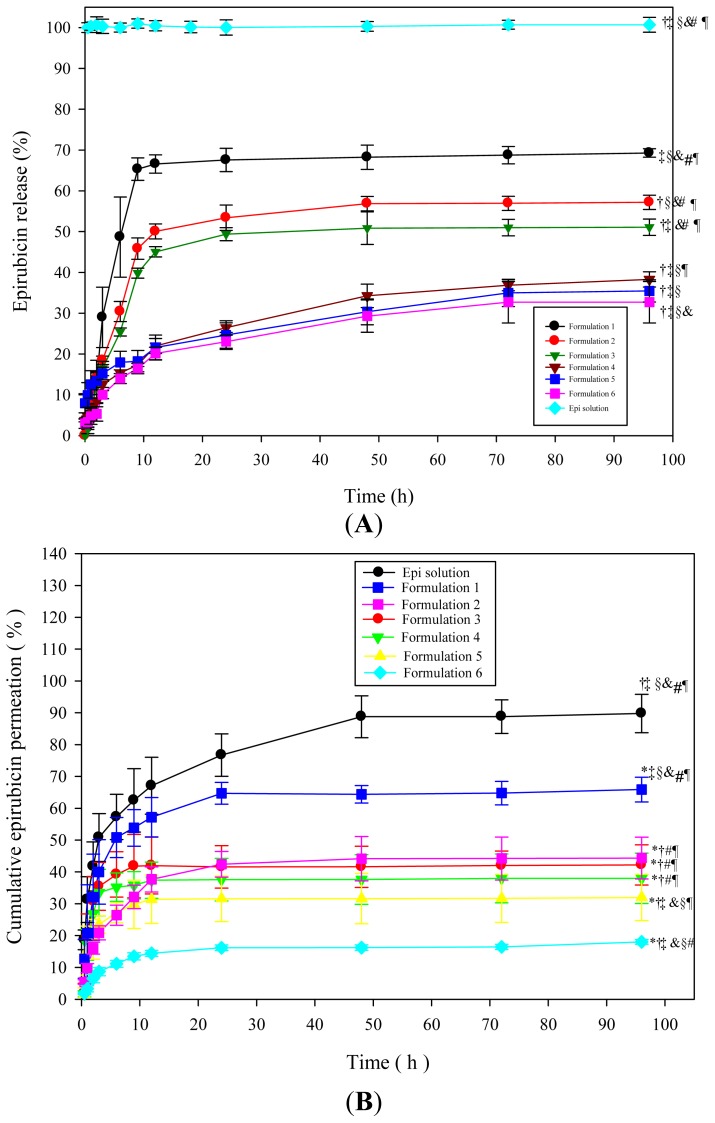

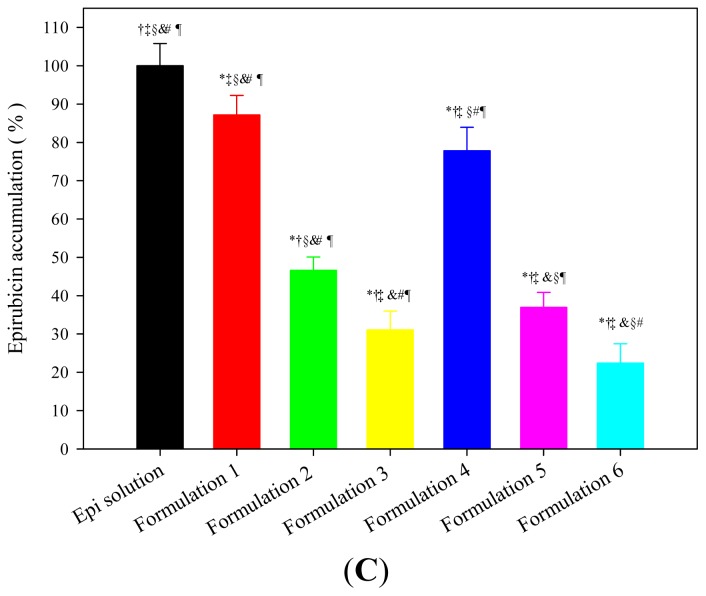
(**A**) *In vitro* release curves of Epi from Formulations 1–6 of liquid and solid suppositories; (**B**) Permeation percentage of Epi from different formulations through the SD rat rectums; and (**C**) Accumulation percentage of Epi from different formulations in the rat rectums. Data shown indicate means ± standard deviation (SD) of six experiments. * *p* < 0.05 compared with Epi solution; ^†^ *p* < 0.05 compared with Formulation 1; ^‡^ *p* < 0.05 compared with Formulation 2; ^§^ *p* < 0.05 compared with Formulation 3; ^&^ *p* < 0.05 compared with Formulation 4; ^#^ *p* < 0.05 compared with Formulation 5; ^¶^ *p* < 0.05 compared with Formulation 6.

The release percentage of Epi from Formulation 1 was significantly higher than those from Formulations 2 and 3 after 96 h of release (*p* < 0.05), as illustrated in Figure 3. Formulations 1–3 of liquid suppository had significantly more release than those of the corresponding Formulations 4–6 of solid suppository, respectively (all *p* < 0.05). This indicates that the presence of cocoa butter in solid suppository may delay Epi release, possibly due to oleaginous property of this base.

#### 2.1.4. *In Vitro* Drug Permeation and Accumulation Tests in Rectal Mucosa

The data in Figure 3B showed that the amount of drugs that penetrated from Epi solution through the rat rectum was higher than those from suppositories. Epi solution not only had the highest permeation percentage but also had the highest accumulation percentage (Figure 3C); but it did not demonstrate a sustained release mode. Liquid suppository in Formulation 1 retained the highest permeation and accumulation percentages among these six Plu/PAA formulations (Figure 3B,C).

#### 2.1.5. FTIR Analysis

**Figure 4.**
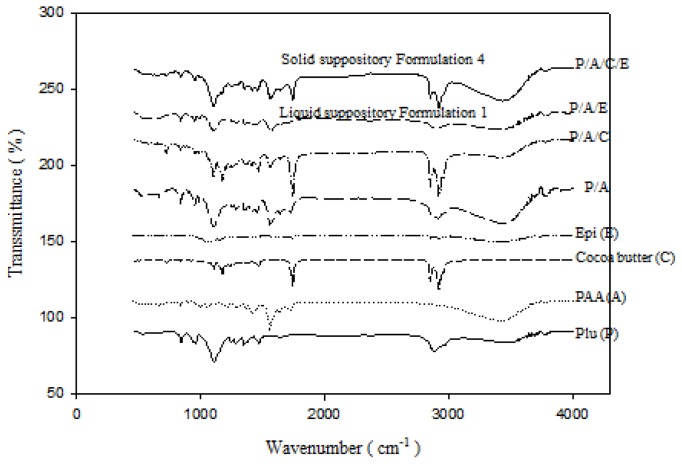
FTIR spectra of Plu (P), PAA (A), cocoa butter (C), and Epi (E), as well as Formulation 1 (P/A/E) and Formulation 4 (P/A/C/E).

Figure 4 exhibits the FT-IR spectra of individual components formulating the liquid and solid suppositories, including Plu, PAA, cocoa butter, Epi, and their mixtures. Figure 4 also shows the FT-IR spectra of Formulation 1 of Plu/PAA/Epi in the liquid suppository and Plu/PAA/cocoa butter/Epi in the solid suppository before storage. As shown in Figure 4, the ether bands of the Pluronic has the absorption peak of C–O–C stretching vibration near the 1111 cm^−1^, CH_2_ stretching vibration at 2898 and 2972 cm^−1^, and O–H stretching at 3448 cm^−1^. Epi, has broad absorption peaks at 3449 cm^−1^ (O–H stretching) and 1638 cm^−1^ (C=O stretching). PAA has absorption peaks of C=O stretching at 1701 cm^−1^ and O–H stretching at 3065 cm^−1^. PAA possesses the characteristic asymmetrical COO^−^ band at 1565 cm^−1^ and symmetrical COO^−^ band at 1472 cm^−1^. The CH_2_ stretching peaks of cocoa butter can be observed in 2895 and 2970 cm^−1^. When the Plu/PAA were mixed, we observed the absorption peaks in spectra at 1652 cm^−1^ (C=O stretching), 1095 cm^−1^ (C–O–C stretching), 2877 and 2985 cm^−1^ (CH_2_ stretching), and 3459 cm^−1^ (O–H stretching). The addition of cocoa butter in the Plu/PAA mixture makes the CH_2_ stretching vibration at 2895 and 2970 cm^−1^ more obvious. The absorption peaks of Epi overlap with those of PAA. There are no additional peaks when Epi was incorporated in the Plu/PAA mixture (liquid suppository) or Plu/PAA/cocoa butter mixture (solid suppository). The absorption peaks of Epi and PAA were combined to form one carbonyl stretching band of 1649 cm^−1^, indicating the formation of hydrogen bonding between carbonyl group of PAA or epirubicin and hydroxyl group of Plu. The characteristic C–O–C stretching band in Plu (originally at 1111 cm^−1^) migrated to 1072 cm^−1^, indicating that the crosslinking of Plu and PAA induced intermolecular hydrogen bonding between Plu and PAA, and thus stabilized the hydrogel mixture.

Both the liquid and solid suppository preparations of Formulations 1 and 4 were stored at 4, 25, and 37 °C for six and 12 months, respectively, and their FTIR spectra are shown in the Supplementary Materials. When we checked the absorption peaks carefully, we observed no obvious changes in the major characteristic spectra for six and 12 months (Figure S1).

#### 2.1.6. *In Vivo* Localization of Suppository in the SD Rats

**Figure 5.**
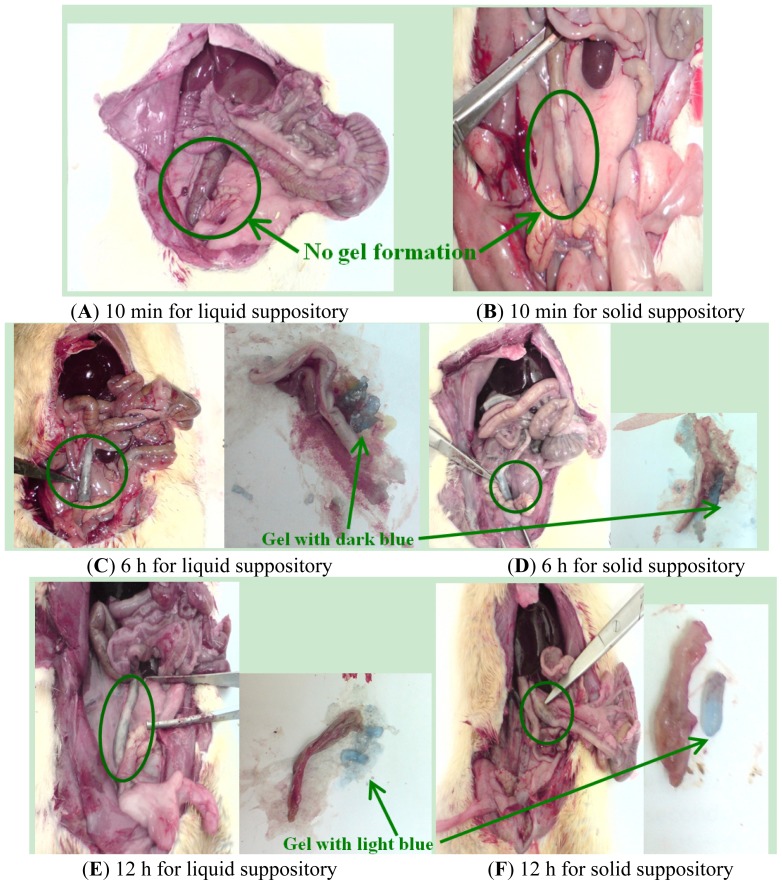
*In vivo* localization of Plu/PAA suppositories in the rectum of SD rats. Plu/PAA liquid and solid suppositories with 0.1% Toluidine Blue O were administered into the anus. (**A**,**B**) 10 min, (**C**,**D**) 6 h, and (**E**,**F**) 12 h after administration of liquid and solid suppositories, respectively, the rectums were sectioned. Note, the gel formation and the color change in gels from dark to light blue after 6 and 12 h of administration.

Liquid suppository and solid suppository with Toluidine Blue O was administered into the rat anus and their retention in the rectum was observed (Figure 5). After administration into the rectum for 10 min, the cocoa butter base of solid suppository melted gradually, but Plu/PAA in solid and liquid suppositories did not form gel immediately. At 6 h after administration, liquid and solid suppositories had similar positions in the lower rectum and formed mucoadhesive hydrogels with a dark blue color. At 12 h after administration, suppositories adhered to the upper rectum in hydrogel form with a light blue color. This means that both liquid and solid suppositories were maintained in the rectum for at least 12 h to sustain release of the enclosed agent.

#### 2.1.7. *In Vivo* Pharmacokinetic Study

**Figure 6.**
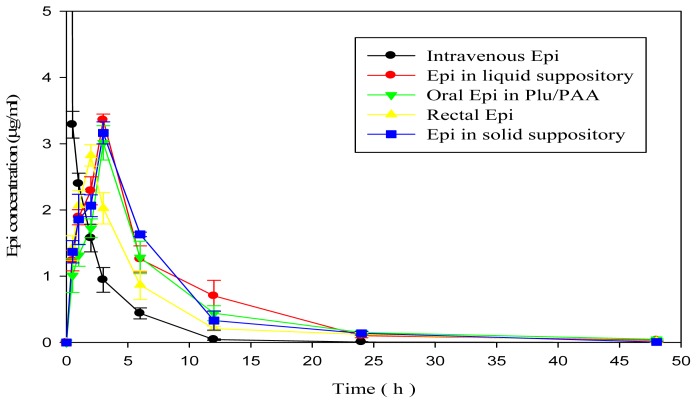
Plasma concentration-time curve of Epi sampled from SD rats after intravenous administration of Epi (50 μg/mL) solution, rectal administration of Epi solution, oral and rectal administration of Epi in Plu 14%/PAA 0.75% liquid or solid suppositories, respectively. Data are means ± SD of six experiments.

The plasma concentration-time curve of Epi sampled from SD rats after administration of different Epi formulations (Groups 1–5) were shown in Figure 6. The corresponding pharmacokinetic parameters were demonstrated in Table 3. The time required to reach peak plasma concentrations (*T*_max_) for liquid and solid suppositories and oral Epi in Plu/PAA (3 h) was 1~3 h later compared with those of rectal Epi solution (2 h) and intravenous Epi solution (0 h), indicating a sustained release effect in the *in situ* hydrogel formulation either in oral or rectal route of administration. The *C*_max_, AUC_0→∞_, and % Fr (relative to intravenous Epi solution) values of Epi in Plu/PAA formulations administered in liquid suppository, solid suppository, or oral routes were higher than that of rectal Epi solution.

#### 2.1.8. Antitumor Efficacy

**Figure 7.**
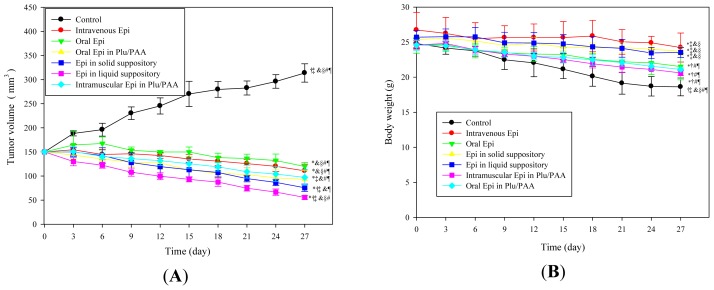
*In vivo* (**A**) antitumor efficacy and (**B**) the body weight (g) of Epi (50 μg/mL) administered in different formulations as a function of time in CT26 bearing mice. For (**A**) and (**B**), data shown indicate mean ± SD of six experiments. * *p* < 0.05 compared with control; ^†^ *p* < 0.05 compared with intravenous Epi; ^‡^ *p* < 0.05 compared with rectal Epi; ^§^ *p* < 0.05 compared with oral Epi in Plu/PAA; ^&^ *p* < 0.05 compared with intramuscular Epi in Plu/PAA; ^#^ *p* < 0.05 compared with Epi in solid suppository; ^¶^ *p* < 0.05 compared with Epi in liquid suppository.

The antitumor efficacy of Epi loaded in different formulations (Groups 1–7) on CT26 bearing Balb/c mice were evaluated for 27 days as shown in Figure 7. Tumors in the saline-treated mice (Group 1) grew continuously throughout the whole 27-day period of treatment. It is noted that Epi administration in different routes inhibited the tumor growth in a similar trend with various degrees (Groups 2–7). We found that the tumor reduction effects of rectal administration of Epi in Plu 14%/PAA 0.75% either in liquid or solid suppository formulations (Groups 6 and 7) were superior to those in all other treatments. In addition, the antitumor efficacy of intramuscular injection of Epi in the Plu 14%/PAA 0.75% formulation (Group 5) was better than that of intravenous Epi solution (Group 2). Oral administration of Epi in Plu 14%/PAA 0.75% (Group 4) also displayed more effectiveness than rectal Epi solution (Group 3) in suppressing tumor growth.

The body weight of mice in Groups 1–5 exhibited a continuous decrease for 27 days due to a loss of appetite as the tumor grew in tumor bearing mice of Groups 1–5, as shown in Figure 7B. However, a marginal body weight reduction, as a sign of treatment safety, was observed in mice treated with rectal Epi in Plu/PAA (Groups 6 and 7).

### 2.2. Discussion

A rectal *in situ* gelling liquid and solid suppository formulations consisting of Plu, PAA, and Epi mixture was evaluated for colorectal cancer therapy. Poloxamer, a FDA-approved non-toxic copolymer, is the most common polymer for formulating a liquid suppository [3,7]. Both the thermogelling and mildly mucoadhesive characteristics make poloxamer-based liquid suppository easy to administrate into the anus, because it is in fluid form at room temperature and becomes a gel at rectal temperature [5,7]. This liquid suppository has been developed as a carrier to incorporate various types of drugs, including fenoterol hydrobromide [3], ibuprofen [24], insulin [25], oxaliplatin [7], and quinine [5]. Furthermore, in the current study, the addition of PAA, a FDA Generally Regarded as Safe (GRAS) excipient, in the suppository formulation markedly improved its mucoadhesion, biodegradation, and gel strength to the rectal mucus membranes and thus prevented leakage. Our previous studies [7,22] have shown that a Plu 14%/PAA 0.75% formulation possesses the advantages, such as suitable gelation temperature, time, and strength, bioadhesive force, water content, swelling ratio, and viscosity for oral and rectal administration. All these characteristics make this formulation more appropriate than those of the gel formed from the individual homopolymers of Plu and PAA, indicating more intense cross-linking networks when Plu interpenetrates with PAA [7,22]. In this study, in addition to Plu 14%/PAA 0.75% (Formulation 1 and 4), we also used Plu 14%/PAA 0.375% (Formulation 2 and 5) and Plu 14%/PAA 0.1875 (Formulation 3 and 6; Table 1) to prepare liquid and solid suppository for *in vitro* studies.

Our current result indicates that the bioadhesive force of the suppository formulation is significantly enhanced with increasing PAA concentration (Table 2). The enhanced bioadhesive force is brought about by the fact that PAA, being a hydrophilic gel-forming bioadhesive polymer, can intensify the bioadhesive force of the suppository formulations [26–28]. Moreover, the addition of cocoa butter to Formulation 1–3 further increases the bioadhesive force of these formulations, as demonstrated in the corresponding bioadhesive force of Formulation 4–6. Cocoa butter, containing a high content of triglycerides derived from stearic, palmitic, and oleic acids, may also reinforce the bioadhesive force of the solid suppositories. Furthermore, because mucin is composed of oligosaccharide chains with sialic acid, Plu with hydroxyl groups and PAA with carboxyl groups may form hydrogen bonds with oligosaccharide chains of mucin, leading to potent bioadhesive forces [29]. Appropriate hydrogel mucoadhesive force is necessary for efficient *in vivo* retention of suppository formulations in a rectal membrane surface for sustained release of incorporated drugs.

We also found an increase in drug release at higher contents of PAA, which has also been reported in other studies [26,27]. When they increased PAA contents in the hydrogel formulation, hydrogel swelling and drug release were increased accordingly. This phenomenon can be attributed to the enhancement in swelling brought about by the additional number of ionizable groups available as the concentration of PAA increases at pH 7.4. This swelling leads to more polymer chain relaxation and thus improved drug release [26,27].

Poloxamer has been found to possess a permeation-enhancing effect after administration of ibuprofen to the mucous membranes and is thus selected as a base for suppositories [24]. The possible explanation for the high permeation and accumulation for Formulation 1 is that more cross-linking between PEO of Plu and carboxylic groups of PAA would allow the swelling hydrogel to become more stretched and more permeable to the incorporated drugs. More sponge-like pores (as shown in scanning electron microscopic images of Lo *et al*. [22]) were thus formed within the hydrogel and would make more Epi diffuse out of the Plu-PAA interpenetrating network of Formulation 1. In the situation of *in vitro* accumulation study, the higher accumulation percentage of Formulation 1 possibly resulted from the relatively more extensive release of Epi, which was sustained in the beaker of the dissolution tester *in vitro*. Furthermore, the protracted exposure of rat rectums to such high concentrations of drugs might intensify the accumulation of Epi in the rectal membrane to achieve a high accumulation percentage. The highest percentage of Epi accumulation in the rectal lumen from Formulation 1 among six Plu/PAA suppository formulations shows a potential of higher percentage of Epi attained in the rectal membranes for long-term release in order to exert their anticancer activity. This is consistent with the better antitumor efficacy observed in the *in vivo* study of CT-26 bearing Blab/c mice. In addition, our FTIR study (Figure 4) also verified that crosslinking of Plu and PAA, as well as their interaction with Epi-induced intermolecular hydrogen bonding among these components, thus stabilized the mixture, as proposed in Figure 1D. Moreover, the major peaks of Formulation 1 at both the liquid and solid suppository preparations did not show remarkable changes at 4, 25, and 37 °C for at least one year (Figure S1), which is a good indication for long-term gel and suppository base evolution. Furthermore, cocoa butter has antioxidant property, which grants it a storage life of two to five years and thus protects the enclosed Plu/PAA/Epi mixture. As summarized from our *in vitro* data, Formulations 1 and 4 possess strong bioadhesive force, long-term appropriate suppository base, sustained release, as well as high accumulation of Epi. We thus selected Formulations 1 and 4 for the following *in vivo* pharmacokinetic and antitumor studies.

The increase in AUC and %Fr for *in situ* hydrogel formulation indicates that Epi oral and rectal absorption was greatly improved by the sustained release gel formed by Plu and PAA. The mucoadhesive property of Plu/PAA polymer to intestinal and rectal membranes may at least partly account for the increase in %Fr of Epi in hydrogels. Furthermore, Plu and PAA may form a gel state and protect the incorporated Epi from degradation and metabolism in the intestine and rectum.

Our finding further indicates that Epi in either solid or liquid suppository formulations exhibited better antitumor efficacy than that of rectal and IV Epi solution in inhibiting CT-26 colon adenocarcinoma cells. We suggest that Epi formulated in Plu/PAA suppository could enhance rectal accumulation of antineoplastic agents in the tumor tissue, possibly caused by sustained release and enhanced permeability and retention (EPR) effects [30]. These effects could be explained by enhanced mucoadhesion, possibly due to the interaction among Plu, PAA, and mucin. The improved transport of Epi from Plu/PAA hydrogels also intensifies passive targeting of Epi through leaky tumor capillary fenestrations into tumor cells by the EPR effect [30,31]. In addition, Plu was found to possess multidrug resistance reversal property in tumors [32] and thus further explained the pronounced inhibition of tumor growth for rectal administration of Epi in Plu/PAA formulations in this study.

We also observed the change in body weight in CT-26 bearing mice as one of the marker of safety. A marginal body weight loss was found in mice treated with rectal Epi in Plu/PAA suppositories (Groups 6 and 7). Consistently, Plu/PAA hydrogels have good compatibility with normal rat intestinal IEC-6 cells and do not damage these normal cells [7]. Moreover, oral Plu/PAA microgels were not absorbed and distributed in blood, liver, or kidney [1], indicating Plu/PAA as safe materials when administered into the body.

## 3. Experimental Section

### 3.1. Materials

Pluronic F-127 (Sigma, St. Louis, MO, USA), acrylic acid (AAc; Fluka, Bornem, Belgium), *N*,*N*,-methylenebisacrylamide (NMBA) (Fluka, Milwaukee, WI, USA), and *N*,*N*,*N*,*N*′-tetramethylethylene-diamine (TEMED) (Fluka, Buchs, Switzerland). Ammonium peroxodisulfate (APS) (Wako, Osaka, Japan). Epirubicin (Pharmorubicin) was purchased from Pfizer (New York, NY, USA). All cell culture media and reagents were purchased from Gibco BRL (Grand Island, NY, USA) or Hyclone (Logan, UT, USA). Most of the other chemical reagents were purchased from either Merck (Darmstadt, Germany) or Sigma-Aldrich (St. Louis, MO, USA). Simulated colorectal fluid (SCF) was prepared as described before [22]. The pH was adjusted to 7.4 to mimic the colorectal pH. Preparation of Epi loaded liquid suppository and solid suppository

### 3.2. Preparation of Epi in Plu/PAA Formulation

#### 3.2.1. Preparation of Plu/PAA Mixture

Three previous studies in our group [7,22,23] have suggested that an optimal Plu concentration for preparing an *in situ* Plu and PAA gelling system used in the intestine and rectum was 14 wt %. Pluronic was slowly added to the distilled water with continuous agitation. It was kept at 4 °C until it became clear solution before use. Various concentration of AAc was added to distilled water to prepare AAc solution with three concentrations of 0.375, 0.75, and 1.5 wt %, respectively. The pH of each solution was adjusted to 4.0 using NaOH or HCl solution. Then, 20 mL of each AAc solution was taken out and 0.2464 g of NMBA (a crosslinking agent), 0.0369 g of APS (an initiator), and 320 μL of TEMED (1.57% *v*/*v*; a promoter) were added to the solution to form PAA solution. Plu and PAA solutions with a volume ratio of 1:1 were mixed in cool bath and stirred for 15 min to obtain the Plu/PAA mixture.

#### 3.2.2. Preparation of Epi in Plu/PAA Liquid Suppository

A calculated amount of Epi was added into Plu/PAA mixture to obtain final Epi concentrations of 25, 50, 75, and 100 μg/mL, and thus forming the liquid suppository.

#### 3.2.3. Preparation of Epi in Plu/PAA Solid Suppository

Cocoa butter was heated to 33 °C. The resulting solution was then placed into the suppository mold with a glass rod inside to obtain the suppository base of 0.5 g. When the suppository base became solid, we slowly added Epi in Plu/PAA mixture, which had been prepared, as mentioned above, to the empty space of the suppository base. The suppository, once filled with the gel mixture, was covered by cocoa butter to close it. The Epi in Plu/PAA solid suppository was cooled down to 25 °C for the following experiments.

### 3.3. Cell Culture

CT26 cells were cultured in Dulbecco’s modified Eagle’s medium (DMEM) supplemented with 10% fetal bovine serum (FBS; Hyclone, Logan, UT, USA), 10 mg/mL streptomycin, and 100 U/mL penicillin (Hyclone, Logan, UT, USA) at 37 °C in an incubator with 5% CO_2_ and 95% air.

### 3.4. Cell Growth Inhibition Assay

Cell viability was determined using an MTT (3-(4,5-dimethylthiazol-2-yl)-2,5-diphenyltetrazolium bromide) assay. The cells were seeded in 96-well plates at a density of 3.5 × 10^4^ cells/well, allowed to attach overnight, and then treated with Plu 14%/PAA 0.75%, Plu 14%/PAA 0.375%, Plu 14%/PAA 0.1875%, or cocoa butter 0.5 g enclosed the above Plu/PAA mixture with various Epi concentrations of 0, 25, 50, 75, and 100 μg/mL for 48 h. At the end of incubation, 100 μL of MTT (0.2 mg/mL) was added and incubated for further 4 h. Then, 100 μL of dimethyl sulfoxide was added to each well to solubilize the formazan. The absorbance was measured at 545 nm using a microplate reader (Dynatech; Chantilly, VA, USA).

### 3.5. Determination of Bioadhesive Force

Male Sprague-Dawley (SD) rats were purchased from the National Laboratory Animal Center (Tainan, Taiwan). The animal-use protocols were in accordance with and permitted by the National University of Tainan Animal Committee. Rat rectums were prepared as described previously [19]. Rats were deprived of food for 1 day and given only sterilized double-distilled water. Fresh rectum was cleaned with SCF and preserved in a refrigerator at 4 °C. The liquid and solid suppository of Formulations 1–6 (Table 1) was cut into 0.1 g sections with a diameter of 0.5 cm and attached to the probes. Each rectum was cut to approximately an area of 2 cm^2^ and placed on the load platform. One hundred milliliters of SCF was then added to the colorectal mucosa. We used Texture Analyzer (TA.XT plus, Stable Micro Systems, Godalming, Surrey, UK) to determine the bioadhesive force at 37 °C and analyze the data. Bioadhesive force *F*_min_, and the detachment stress (g), was measured as the minimal weight that detached colorectal mucosa and the indicated formulation. The test parameters were set as described previously [22].

### 3.6. *In Vitro* Drug Release Study

Epi release from liquid and solid suppository of three formulations (Table 1) was measured using a dissolution tester (DT-6, Hsiangtai, Changhua, Taiwan) according to an *in vitro* modified dissolution test comprising the USP Paddle Method. The lab-made suppository containers were filled with weighed Epi suppository of six formulations and covered with mesh size plastic nets, respectively. These preparations were immersed in 400 mL of SCF (pH = 7.4) at 37 °C, which was stirred at a rate of 100 rpm for maintaining a uniform drug concentration in the medium. At specific time intervals, the samples of 1 mL were removed and stored at −20 °C until analysis. With each sampling, the medium was replaced with pre-warmed SCF to maintain the total volume constant. The release of Epi from the suppository formulations was sampled up to 96 h. The samples were centrifuged at 13,000 rpm for 10 min. The concentration of Epi in the collected supernatants was determined by HPLC. All the release experiments were repeated six times.

### 3.7. Epi Concentration Analyzed Using HPLC

The analytical method for Epi was modified from previous reports [19]. The HPLC system consisted of a pump (L7100, Hitachi, Tokyo, Japan) equipped with an automated injector (L2200), a 5 μm LiChrospher column (Merck, Whitehouse, NJ, USA) and a UV detector (L2400). The mobile phase included methanol and water (75:25, *v*/*v*), run at a flow rate of 1.2 mL/min. The detection wavelength was 254 nm.

### 3.8. *In Vitro* Drug Penetration Test

SD rats bred and housed in the animal center were used. Rat rectums were prepared using a method previously described [19]. Suppository formulations (Table 1; 9 mL each) were administered into a section of rat rectum (8 cm), respectively. Both sides of the rat rectum were tied up with a thread to prevent leakage. The rat rectum was then placed in a dissolution tester (DT-6, Hsiangtai, Changhua, Taiwan). Epi permeation test was performed at 37 °C at 100 rpm with 400 mL of simulated colorectal fluid (pH 7.4) as permeation media. At predetermined interval, 200 μL of the medium was sampled and stored at −20 °C until analysis. The permeation media were replaced by pre-warmed fresh simulated colorectal fluid. The filtrate was analyzed for Epi by HPLC (L7100, Hitachi), as described above. Six measurements from each formulation were analyzed.

### 3.9. *In Vitro* Drug Accumulation Study

After finishing the *in vitro* drug permeation test, the rat rectum was removed and the mucosa was washed by phosphate buffer (pH 7.4) to remove any residual suppository and Epi. The rat rectum was minced, extracted with methanol and SCF (1:1, *v*/*v*), and then centrifuged. The supernatant layer was analyzed for Epi by HPLC. Six samples from each formulation were measured.

### 3.10. FT-IR Analysis

Plu/PAA/Epi liquid and solid suppository formulations and their corresponding components were stored at 4, 25, and 37 °C and sampled at predetermined time points of 0, 6, and 12 months, respectively. The samples were dried completely by a freeze dryer (FDU-1200, EYELA, Tokyo, Japan). The dried powders were mixed with KBr powder at a ratio of 1:99 with careful grinding in an agate motor and pressed into a slice. The FTIR spectra were recorded using a Lambda 25 Fourier transform infrared spectrometer (PerkinElmer, Inc., Waltham, MA, USA).

### 3.11. Identification of Suppository Localization *in Vivo*

Male SD rats weighing about 450 g fasted for 24 h. before the experiment, but allowed free access to water. One milliliter of Plu 14%/PAA 0.75% solution with 0.1% Toluidine Blue O (liquid suppository) or cocoa butter base enclosing the above mixture (solid suppository) were administered into the rectum 2 cm above the anus. At 10 min, 6 h, and 12 h after administration, rectum was sectioned and the status and localization of suppository in the rectum were identified by the blue color.

### 3.12. *In Vivo* Pharmacokinetic Study

Male SD rats weighing approximately 400 g were fasted for 24 h prior to the experiments but were allowed free access to water. The rats were randomly assigned to five groups (*n* = 6 for each group): Group 1 treated with intravenous Epi solution (50 μg/mL, 1 mL), Group 2 for rectal Epi solution, Group 3 for oral Epi in Plu14%/PAA0.75%, Group 4 for rectal liquid suppository of Epi in Plu14%/PAA0.75%, Group 5 for rectal solid suppository (cocoa butter base enclosing Epi in Plu14%/PAA0.75%). Blood samples of 100 μL were taken at indicated intervals for 48 h from the tail vein and the plasma concentration of Epi in the samples was analyzed by HPLC. *T*_max_(h), *C*_max_(μg/mL), and AUC_0→∞_ (h × μg/mL) were then estimated and calculated. Six measurements from each formulation were analyzed. Relative bioavailability (%Fr) of Epi formulations was calculated using the following formula:

(1) $$\%\text{Fr}=({\text{AUC}}_{\text{other}}\times {\text{D}}_{\text{iv}})/({\text{AUC}}_{\text{iv}}\times {\text{D}}_{\text{other}})\times 100$$

### 3.13. Establishment of *in Vivo* Model of Mouse Tumor Xenografts

Balb/c mice, 6 weeks old, were purchased from BioLasco (Taipei, Taiwan) and maintained in the individual ventilation cage system. The animals had free access to sterilized food and water. Then, CT26 (10^6^ cells) in 0.1 mL PBS were inoculated subcutaneously into the right flank of the mouse. The tumor grew in the right flank area near the back of the mouse. All of the procedures performed on animals have been approved by the National Tainan University Animal Committee.

### 3.14. *In Vivo* Antitumor Efficacy

When the tumor volume reached approximately 150 mm^3^ in size (calculated according to Equation 2), the mice were randomly assigned to seven groups (*n* = 6 for each group): Group 1 treated with rectal saline solution (control), Group 2 for intravenous Epi solution (50 μg/mL, 150 μL), Group 3 for rectal Epi solution, Group 4 for oral Epi in Plu14%/PAA0.75%, Group 5 for intramuscular Epi in Plu14%/PAA0.75%, Group 6 rectal liquid suppository of Epi in Plu14%/PAA0.75%, Group 7 for rectal solid suppository (cocoa butter base enclosing Epi in Plu14%/PAA0.75%). Mice were administered by injection, oral, or rectal routes every 3 days for a total of 27 days. Animal body weight and tumor size were measured every 3 days for 27 days. Changes in tumor volume were used as an overall indicator for antitumor efficacy. Tumor size was measured with a digital caliper, and the tumor volume (V) was calculated using the formula:

(2) $$V=(L\times W^{2})/2$$

where length (L, mm) is the longest diameter and width (W, mm) is the shortest diameter perpendicular to length.

### 3.15. Statistical Analysis

Results are given as means ± SD. Statistical analysis was performed using Student’s *t*-test. Multiple comparisons were done using one way ANOVA and Dunnett’s test. Statistical significance was set at *p* < 0.05.

## 4. Conclusions

Taken together, our current findings show that Epi/Plu/PAA in solid and liquid suppository formulations inhibited colorectal tumor growth with reduced weight loss. Pluronic and PAA were found to be nontoxic but increased chemosensitization of colon cancer cells to Epi. These suppositories were administered into the rectum of rats without difficulty and leakage, and remained in the rectums of fasted rats for at least 12 h. This study highlights the benefits of using pH- and thermo-sensitive hydrogel to deliver Epi. These benefits include long-term appropriate suppository base and high accumulation of Epi in the rectum with the characteristics of sustained-release and mucoadhesion. Our results provide the foundation for future development of long-acting *in situ* Plu/PAA hydrogels for treatment of colon cancer in a convenient suppository formulation.

## Supplementary Information



## Figures and Tables

**Table 1.** Epirubicin (Epi) in Pluronic (Plu)/polyacrylic acid (PAA) hydrogels of both liquid and solid suppository formulations.

Hydrogel formulations	Composition (wt %)	
Formulation 1	Plu 14%/PAA 0.75%/Epi 50 μg/mL	Liquid suppository
Formulation 2	Plu 14%/PAA 0.375%/Epi 50 μg/mL	
Formulation 3	Plu 14%/PAA 0.1875%/Epi 50 μg/mL	

Formulation 4	Cocoa butter 0.5 g/Plu 14%/PAA 0.75%/Epi 50 μg/mL	Solid suppository
Formulation 5	Cocoa butter 0.5 g/Plu 14%/PAA 0.375%/Epi 50 μg/mL	
Formulation 6	Cocoa butter 0.5 g/Plu 14%/PAA 0.1875%/Epi 50 μg/mL	

**Table 2.** Bioadhesive force of suppository formulations.

Suppository formulations	*F*max (g)
Formulation 1	6.70 ± 0.23
Formulation 2	5.39 ± 0.27
Formulation 3	3.78 ± 0.13
Formulation 4	6.99 ± 0.25
Formulation 5	5.64 ± 0.21
Formulation 6	4.05 ± 0.29
Cocoa butter	5.94 ± 0.57

**Table 3.** The pharmacokinetic parameters of Epi administered in different formulations and routes into SD rats.

Formulations	*C*_max_ (μg/mL)	*T*_max_ (h)	AUC_0→∞_ (h × μg/mL)	%Fr
Intravenous Epi	100.00 ± 0.00 †,‡,§,&	0	34.38 ± 0.85 †,‡,§,&	100.00 ± 2.47 †,‡,§,&
Rectal Epi	2.81 ± 1.38 *	2	17.26 ± 1.95 *,‡,§,&	50.20 ± 5.67 *,‡,§,&
Oral Epi in Plu/PAA	3.01 ± 1.08 *	3	22.85 ± 2.04 *,†	66.46 ± 5.93 *,†
Epi in liquid suppository	3.36 ± 2.17 *	3	23.04 ± 2.37 *,†	67.02 ± 6.89 *,†
Epi in solid suppository	3.16 ± 1.18 *	3	22.14 ± 2.07 *,†	64.40 ± 6.02 *,†

* *p* < 0.05 compared with Intravenous Epi;

† *p* < 0.05 compared with Rectal Epi;

‡ *p* < 0.05 compared with Oral Epi in Plu/PAA;

§ *p* < 0.05 compared with Epi in liquid suppository;

& *p* < 0.05 compared with Epi in solid suppository.
