# Supplementary material for: Evaluation of Epirubicin in Thermogelling and Bioadhesive Liquid and Solid Suppository Formulations for Rectal Administration
Source: Int J Mol Sci. 2013 Dec 31;15(1):342–60. doi: 10.3390/ijms15010342 (PMC3907813; doi:10.3390/ijms15010342)
Supplement: Supplementary file 1 [file ijms-15-00342-s001.pdf]

Supplementary Information

Figure S1. FTIR spectra of (**A**) Formulation 1 for 6 months; (**B**) Formulation 1 for 12 months; (**C**) Formulation 4 for 6 months; and (**D**) Formulation 4 for 12 months. These preparations were stored at (**a**) 4 °C, (**b**) 25 °C, and (**c**) 37 °C.

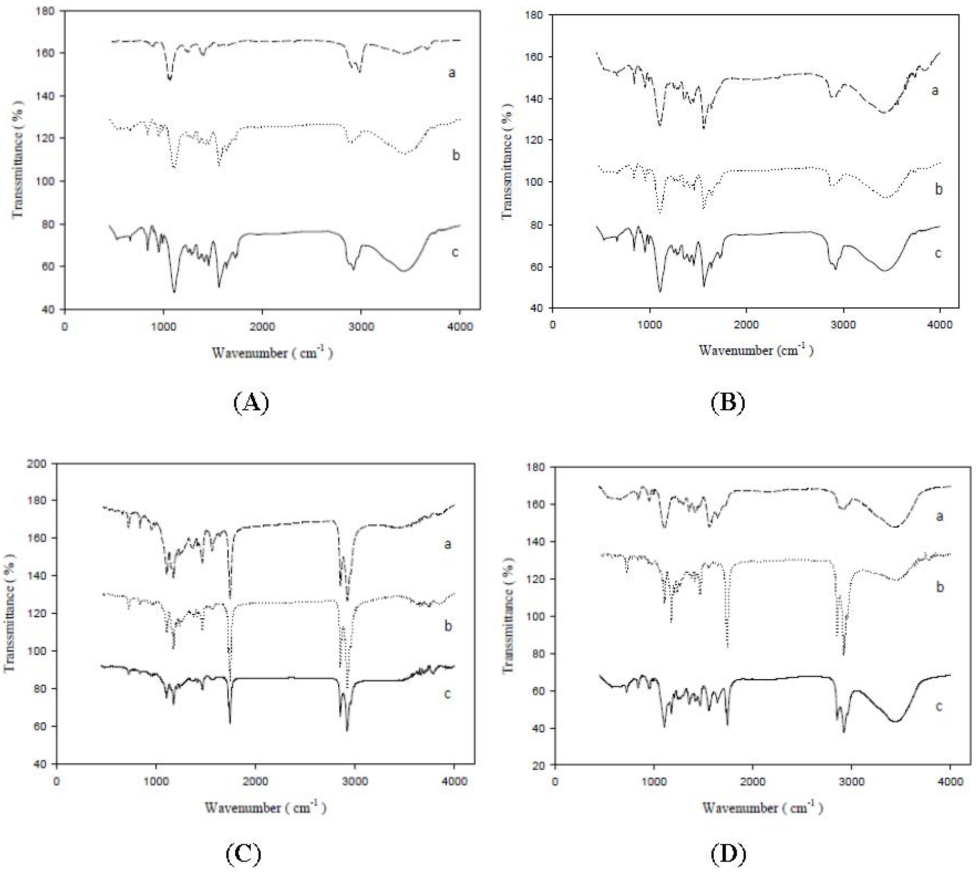

© 2013 by the authors; licensee MDPI, Basel, Switzerland. This article is an open access article distributed under the terms and conditions of the Creative Commons Attribution license (http://creativecommons.org/licenses/by/3.0/).
